# Serum extracellular traps associate with the activation of myeloid cells in SLE patients with the low level of anti-DNA antibodies

**DOI:** 10.1038/s41598-022-23076-1

**Published:** 2022-11-01

**Authors:** Norio Hanata, Mineto Ota, Yumi Tsuchida, Yasuo Nagafuchi, Tomohisa Okamura, Hirofumi Shoda, Keishi Fujio

**Affiliations:** 1 Department of Allergy and Rheumatology, Graduate School of Medicine, The University of Tokyo, Tokyo, Japan; 2 Department of Functional Genomics and Immunological Diseases, Graduate School of Medicine, The University of Tokyo, Tokyo, Japan

**Keywords:** Rheumatology, Rheumatic diseases, Systemic lupus erythematosus

## Abstract

Neutrophil extracellular traps (NETs) are involved in systemic lupus erythematosus (SLE). We sought to cluster SLE patients based on serum NET levels. Serum NET levels were higher in SLE patients than healthy controls. Frequencies of pleuritis and myositis were increased in patients with high serum NET levels. Serum NET levels negatively correlated with anti–double stranded DNA (anti-dsDNA) antibody titers and C1q-binding immune complexes, but positively correlated with C-reactive protein (CRP) and monocyte counts. Neutrophil transcriptome analysis demonstrated no difference in NET-associated signatures, irrespective of serum NET levels, suggesting anti-dsDNA antibody-mediated clearance of NETs. In serum, NET levels were significantly correlated with myeloid cell-derived inflammatory molecules. Serum NET-based cluster analysis revealed 3 groups of patients based on serum NET and CRP levels, anti-dsDNA antibody titers, and monocyte count. Monocytes were consistently activated following NET-containing immune complex (NET-IC) stimulation. In conclusion, SLE patients with high serum NET levels had lower anti-dsDNA antibody titers and higher inflammatory responses. NET-IC-stimulated monocytes might associate with an inflammatory response characterized by elevated CRP levels. These findings can apply to precision medicine, as inflammatory processes, rather than antibody-dependent processes, can be targeted in specific subpopulations of SLE patients.

## Introduction

Systemic lupus erythematosus (SLE) is an autoimmune disease, and several immunological processes take part in its pathogenesis. SLE is clinically and immunologically heterogenous. Some patients manifest with higher serum anti-double stranded DNA (anti-dsDNA) antibody levels, and immune complex-dependent processes which contribute to vascular and organ damage, such as lupus nephritis^1^. Other patients have a more inflammatory phenotype with fever, arthritis^2^, serositis^3^, and myositis^4^. Given the differences in clinical presentation and pathobiological mechanisms of SLE, it would be desirable to have a personalized medicine approach to therapy, and to achieve this, an understanding of the heterogeneity of SLE pathogenesis is required.

Neutrophil extracellular traps (NETs) are released from activated neutrophils^5^. NETs are hypothesized to have a pathogenic role in SLE as they contain autoantigens, such as LL37, myeloperoxidase (MPO), and histones^6^. NETs also contain nucleic acids, which activate Toll-like receptors^7^ and the cGAS-STING pathway^8^, driving proinflammatory processes. NETs also play a pivotal role in thrombosis^9^. Serum NET levels can be measured by detecting MPO-DNA complexes or citrullinated histones, and an elevation of circulating NETs has been observed in several autoimmune and infectious diseases^10^. Additionally, we have reported that peptidylarginine deiminase 4 (PAD4) deficiency ameliorates disease in a lupus mouse model^11^. As NET formation was clearly suppressed in these *Padi4*-deficient mice, the lack of NETs was thought to be one of the reasons for the ameliorated lupus phenotype. Recent studies implicate NETs as key players in SLE pathogenesis^12^, and focusing on circulating NETs (mostly NETs, and might also be monocyte and macrophage extracellular traps (METs)^13^), which can be measured by MPO-DNA complex levels, can be an important clue for patient stratification prior to treatment. However, it is currently unclear if circulating NETs could be of use as a clinical biomarker to stratify SLE patients.

Recently, we established the Immune Cell Gene Expression Atlas from the University of Tokyo (ImmuNexUT) database, which consists of peripheral blood immune cell transcriptomes and whole genome sequence data from 337 patients with immune-mediated diseases and 79 healthy volunteers^14^. Using this database, SLE patients can be clustered into subpopulations based on transcriptional modules and immunological phenotypes.

Here, serum MPO-DNA complex levels were measured in SLE patients. The aim of this study was to cluster SLE patients based on clinical, immunological, and transcriptomic parameters, and to correlate these with serum NET levels in order to identify a biomarker that could potentially be used to individualize treatment.

## Materials and methods

### Participants

SLE patients who were admitted to the University of Tokyo Hospital from September 2017 to June 2020 were enrolled. All patients fulfilled the American College of Rheumatology (ACR) 1997 classification criteria^15^ and the Systemic Lupus International Collaborating Clinics (SLICC) 2012 criteria^16^ for SLE. Patients with malignancy or acute infectious disease were excluded. Clinical and demographic information, such as laboratory data and SLE disease activity index 2000 (SLEDAI-2K) scores and their components were extracted from medical records. High disease activity was defined SLEDAI-2K > 10 as previously described^17^. Age- and sex-matched healthy controls (HCs) were also recruited. All patients and HCs were Japanese. We obtained written informed consent from all participants. This study and the methods of this study were approved by the ethical committees of the University of Tokyo Hospital (11592 and G10095) and were performed in accordance with the latest version of the Helsinki declaration.

### Serum MPO-DNA complex measurement

Serum MPO-DNA complex levels of SLE patients and HCs were measured as previously reported^18^. In brief, high-binding 96-well microplates (Corning) were incubated overnight at 4 °C with a mouse anti-human MPO antibody (clone 4A4; Bio-Rad) diluted in coating buffer (Cell Death Detection ELISA kit; Roche). Following blocking with 1% bovine serum albumin (cat# A2153; Sigma) in phosphate-buffered saline (PBS), plates were incubated at room temperature with 10% human serum in blocking buffer, washed, and then 10 × anti-DNA-POD (Clone MCA-33, Cell Death Detection ELISA kit; Roche) in blocking buffer was added. After the incubation, TMB substrate (KPL) was added, and absorbance was measured at 450 nm after the addition of the stop reagent (Wako). Serum MPO-DNA complex “high” patients were defined as those whose optical density values were two standard deviations higher the mean of HCs.

### Measurement of serum cytokines and functional proteins

Serum cytokines and functional proteins were measured by Luminex assays (R&D Systems).

### Measurement of clinical parameters from patient blood sample

For complete blood cell count, electrical resistance method and a flow cytometric method were used, and their differential was evaluated by XN-Series (Sysmex). A latex aggregation test was used for C-reactive protein measurement, and C1q-binding immune complexes levels were evaluated by ELISA. C3 and C4 were quantified by immunonephelometric assays and CH50 was measured by liposome immunoassay. Anti-dsDNA antibodies were measured by fluoroenzyme immunoassay (FEIA) method, with the manufacturer’s cut-off value of 10 U/mL.

### Transcriptome data analysis

Transcriptome data of neutrophils and monocytes in HCs and SLE patients were retrieved from the ImmuNexUT database^14^. In this database, neutrophils were isolated using the MACSxpress Neutrophil Isolation Kit (Miltenyi Biotec). The detailed sampling protocol has been described elsewhere^14^.

Differential expression analysis was performed with edgeR after normalization using trimmed mean of M values. Differentially expressed genes (DEGs) were detected with a false discovery rate (FDR) of < 0.05. The NET score, interferon sore, immature neutrophil score, chemotaxis score, and phagocytosis score were calculated by the Gene Set Variation Analysis (GSVA) using previously reported gene sets^14,19,20^.

### Induction and collection of NETs and immune complex formation for in vitro monocyte stimulation

Neutrophils from SLE patients were isolated using Polymorphprep (Axis-Shield) and were resuspended with HBSS(-) (Wako) supplemented with 0.05% fetal bovine serum (FBS), 10 mM HEPES (Thermo Fisher Scientific), and 1 mM CaCl_2_ (Wako). Neutrophils were seeded at a density of 2 × 10^6^ cells/mL and were stimulated with 25 µM calcium ionophore (Sigma-Aldrich) for 4 h at 37 °C in 5% CO_2_. Then, the media was removed and cells were washed twice with prewarmed PBS. NETs were digested with 15 U/mL of Micrococcal Nuclease (Thermo Fisher Scientific) for 20 min at 37 °C in 5% CO_2_. Supernatants were collected and centrifuged for 5 min at 300 g and stored at − 30 °C. The concentration of ds-DNA in NETs was measured using the Qubit dsDNA HS Assay Kit (Thermo Fisher Scientific).

Serum IgG was purified from HC and SLE patients with a serum concentration of anti-dsDNA higher than 90 IU/mL using HiTrap Protein G HP columns (GE Healthcare Life Sciences). Following desalination using slide-A-LyzerTM G2 Dialysis Cassettes (Thermo Fisher Scientific), antibody concentrations were measured using the Pierce BCA Protein Assay Kit (Thermo Fisher Scientific). For immune complex formation, NETs from SLE patients and serum IgG from HCs or SLE patients were mixed and incubated for 1 h at 37 °C, as previously reported^21^.

### Monocyte stimulation with NETs or an immune complex

Peripheral blood mononuclear cells were isolated from HC by density gradient separation with Ficoll-Paque PLUS (GE Healthcare). Following erythrocyte lysis with potassium ammonium chloride buffer, monocytes were isolated with CD14 microbeads (Miltenyi Biotec). Monocytes were seeded at a density of 1 × 10^6^ cells/mL in a medium of RPMI 1640 supplemented with 10% FBS, 100 µg/mL L-glutamine (Sigma), 100 U/mL penicillin, and 100 µg/mL streptomycin (Sigma). They were then stimulated with 300 ng/mL of SLE patient–derived NETs or with an immune complex of NETs plus IgG from SLE patients or HCs (SLE NETs-SLE IgG [10 µg/mL] or SLE NETs-HC IgG [10 µg/mL]) for 2 days at 37 °C in 5% CO_2_. Some wells were preincubated for 2 h at 37 °C in 5% CO_2_ with 1000 IU/mL IFNα-2b (MSD), as previously reported^21^. Following incubation with NETs or an immune complex, monocyte activation was evaluated by the proportion of activated monocytes (7AAD^-^CD14^+^CD86^+^HLA-DR^+^) using an 8-color MoFlo XDP (Beckman Coulter). Data were analyzed using FlowJo Software (https://www.flowjo.com/index.php) (Tree star).

Antibodies used for cellular staining were as follows: Human Fc Receptor Binding Inhibitor Purified (eBioscience), CD14-FITC (M5E2, BioLegend), CD86-APC (IT2.2, BioLegend), HLA-DR-APC-Cy7 (L243, BioLegend), and 7-AAD (BioLegend).

### Statistics

GraphPad Prism 9.4.0 (https://www.graphpad.com/scientific-software/prism/) (GraphPad Software) and JMP Pro 15 (SAS Institute Inc.) were used for all statistical analyses, except for RNA sequencing data. RNA sequencing data was analyzed using R version 3.5.0 (R Foundation for Statistical Computing). Categorical data were tested with Fisher’s exact tests. Normality was evaluated using Shapiro–Wilk test. For normally distributed continuous data, two-tailed unpaired t tests were used to compare between two groups, and one-way ANOVAs with Tukey’s correction for multiple comparisons were used to compare among three or more groups. Differences between two groups of non-normally distributed continuous data were tested for significance with nonparametric Mann–Whitney *U* tests. Correlations were evaluated by nonparametric Spearman’s rank correlation coefficients. *P* values < 0.05 were considered significant.

## Results

### Clinical characteristics of SLE patients and serum MPO-DNA complex levels

**Figure 1:**
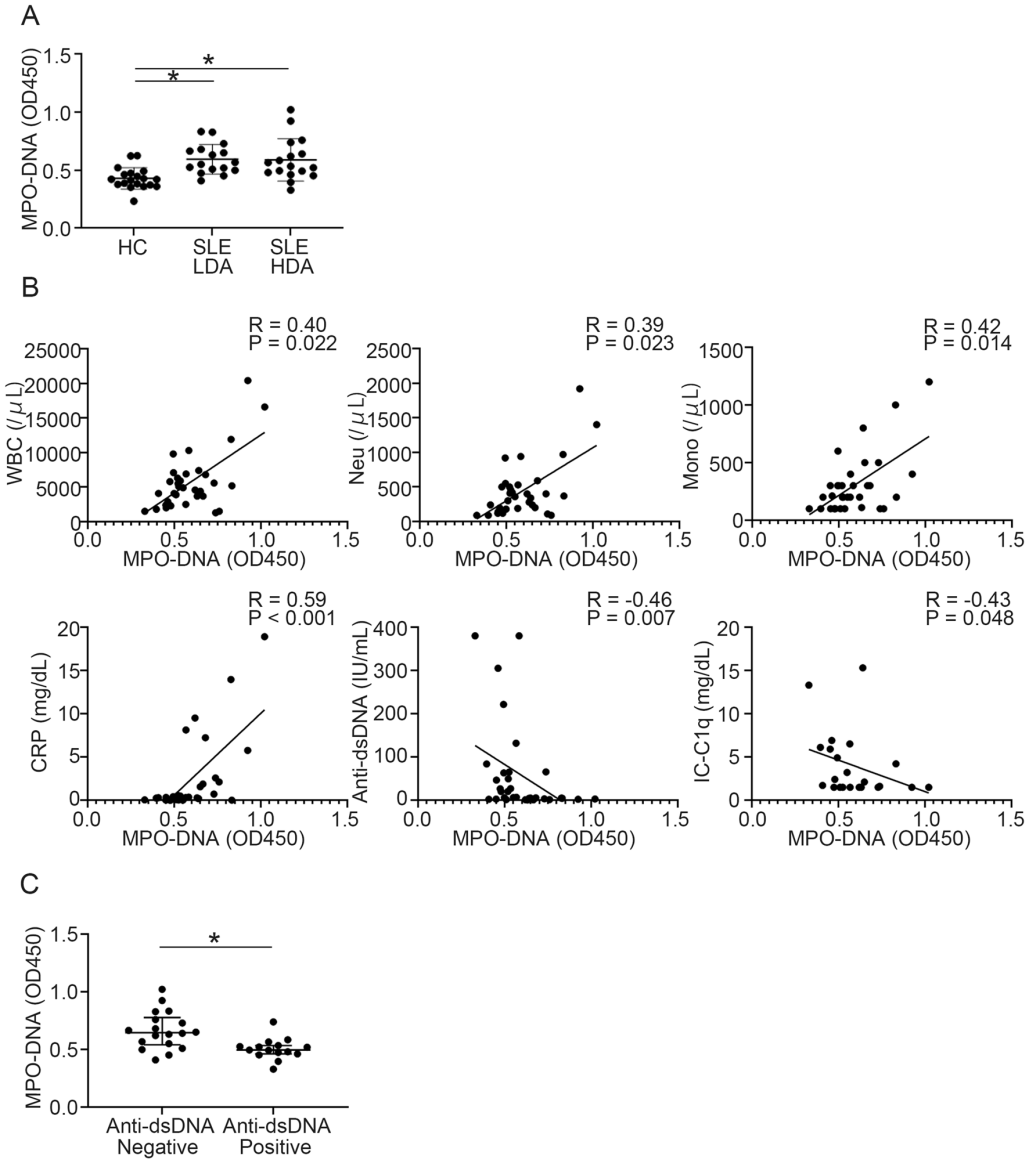
Serum MPO-DNA complex levels and their correlations with clinical parameters in SLE patients. (**A**) Serum MPO-DNA complex levels in HCs (n = 19) and SLE patients with low disease activity (n = 16) and high disease activity (n = 17). *P < 0.05, one-way ANOVA with Tukey’s multiple comparisons test. Patients with high disease activity was defined SLEDAI-2K > 10. (**B**) Correlations between serum MPO-DNA complex levels and clinical parameters, including white blood cell, neutrophil, and monocyte counts; CRP and IC-C1q levels; and anti-dsDNA antibody titers in SLE patients. n = 33 for each clinical parameter, except for IC-C1q levels (n = 22); Spearman’s rank correlation coefficients. (**C**) Serum levels of MPO-DNA were compared between anti-dsDNA antibody-positive (n = 15) and -negative (n = 18) patients. *P < 0.05; Mann–Whitney *U* test. Error bars represent median ± IQR. Anti-dsDNA: anti-double stranded DNA antibody; CRP; C-reactive protein; HC: healthy controls; IC-C1q: C1q-binding immune complexes; HDA: high disease activity; LDA: low disease activity; Mono; monocyte; Neu: neutrophil; SLE; systemic lupus erythematosus; WBC: white blood cell.

Thirty-three SLE patients, and 19 age- and sex-matched HCs were enrolled. Demographic and clinical information is summarized in Supplementary Table S1. About half of the SLE patients had severe disease activity, and the mean (SD) SLEDAI-2K score in SLE patients was 12.36 (7.63). As reported previously^22^, serum MPO-DNA complex levels were significantly elevated in SLE patients both with low and high disease activity compared with HCs (Fig. 1A). Since we hypothesized that serum MPO-DNA complex levels could be a biomarker of specific lupus disease manifestations, we correlated serum MPO-DNA complex levels with several blood tests readily available in the clinical setting. Serum MPO-DNA complex levels positively correlated with white blood cell, neutrophil, and monocyte counts and C-reactive protein (CRP) levels, whereas they negatively correlated with anti-dsDNA antibody titers and C1q-binding immune complexes (Fig. 1B, Supplementary Table S2). Statistical significance was observed in CRP and anti-dsDNA antibody titers after multivariate analysis (Supplementary Table S2). Indeed, SLE patients with high levels of serum MPO-DNA complex showed significantly lower positivity of anti-dsDNA antibody titers and higher levels of CRP (Table 1). However, there was no correlation between serum MPO-DNA complex levels and SLEDAI-2K score (Supplementary Table S2), and there was no significant difference in SLEDAI-2K score between serum MPO-DNA complex high and low groups (Table 1). The presence of other autoantibodies (anti-SSA, anti-SSB, anti-RNP, anti-Sm, anti-cardiolipin β2GPI antibodies) and lupus anticoagulant was identical, regardless of the serum MPO-DNA complex levels (Supplementary Table S3). Notably, the frequencies of myositis and pleurisy were significantly higher in SLE patients who had high serum MPO-DNA than in those with low levels (Table 1) while the frequency of other clinical manifestations were not dependent on MPO-DNA complex levels. In addition, serum levels of the MPO-DNA complex were significantly higher in SLE patients who were anti-dsDNA antibody-negative (Fig. 1C, Supplementary Table S4). Moreover, anti-dsDNA antibody-negative patients had lower SLEDAI-2K scores, higher lymphocyte and monocyte counts, and higher serum levels of CH50 and C3 compared with anti-dsDNA antibody-positive patients (Supplementary Figs. S1A, S1B, Supplementary Table S4). Therefore, SLE patients with high serum levels of the MPO-DNA complex were characterized by a higher frequency of myositis and pleurisy, higher serum level of CRP, and lower titers of anti-dsDNA antibodies. In contrast, anti-dsDNA antibody-positive patients showed lower serum levels of the MPO-DNA complex and CRP.

**Table 1:** Comparison of patients with and without high MPO-DNA complex levels in each SLEDAI-2K variable.

	MPO-DNA low (n = 20)	MPO-DNA high (n = 13)	*P* value
Organic brain syndrome (%)	1/20 (5.00)	0/13 (0.00)	1.00
Visual disturbance (%)	3/20 (15.00)	1/13 (7.69)	1.00
Cranial nerve disorder (%)	1/20 (5.00)	0/13 (0.00)	1.00
Lupus headache (%)	2/20 (10.00)	0/13 (0.00)	0.51
Vasculitis (%)	2/20 (5.00)	0/13 (0.00)	0.51
Arthritis (%)	10/20 (50.00)	5/13 (38.46)	0.72
Myositis (%)	1/20 (5.00)	5/13 (38.46)	0.025
Urinary casts (%)	5/20 (25.00)	2/13 (15.38)	0.68
Hematuria (%)	6/20 (30.00)	0/13 (0.00)	0.060
Proteinuria (%)	5/20 (25.00)	3/13 (23.08)	1.00
Pyuria (%)	3/20 (15.00)	1/13 (7.69)	1.00
Rash (%)	2/20 (10.00)	4/13 (30.77)	0.18
Alopecia (%)	2/20 (10.00)	1/13 (7.69)	1.00
Mucosal ulcers (%)	2/20 (10.00)	2/13 (15.38)	1.00
Pleurisy (%)	1/20 (5.00)	5/13 (38.46)	0.025
Pericarditis (%)	0/20 (0.00)	2/13 (15.38)	0.15
Low complement (%)	17/20 (85.00)	8/13 (61.54)	0.21
Increased anti-dsDNA Ab (%)	14/20 (70.00)	1/13 (7.69)	< 0.001
Fever (%)	5/20 (25.00)	5/13 (38.46)	0.46
Thrombocytopenia (%)	2/20 (10.00)	4/13 (30.77)	0.18
Leukopenia (%)	4/20 (20.00)	2/13 (15.38)	1.00
SLEDAI-2 K score (mean [SD])	13.95 (7.43)	9.92 (7.57)	0.14
CRP (mg/dL; median [IQR])	0.22 (0.033–0.35)	2.10 (0.49–8.37)	0.001
ESR (mm/h; mean [SD])	48.55 (30.25)	62.85 (34.04)	0.22

The MPO-DNA high group was defined as OD value > 0.62 (mean + 2SD of HC). Group differences in the proportion of categorical variables were analyzed using Fisher’s exact tests.

*Anti-dsDNA Ab* anti-double stranded DNA antibody, *CRP* C-reactive protein, *ESR* erythrocyte sedimentation rate, *HC* healthy control, *MPO-DNA* myeloperoxidase-DNA, *OD* optical density, *SD* standard deviation, *SLEDAI-2K* systemic lupus erythematosus disease activity index 2000.

### Transcriptomic analysis of neutrophils in SLE patients

**Figure 2:**
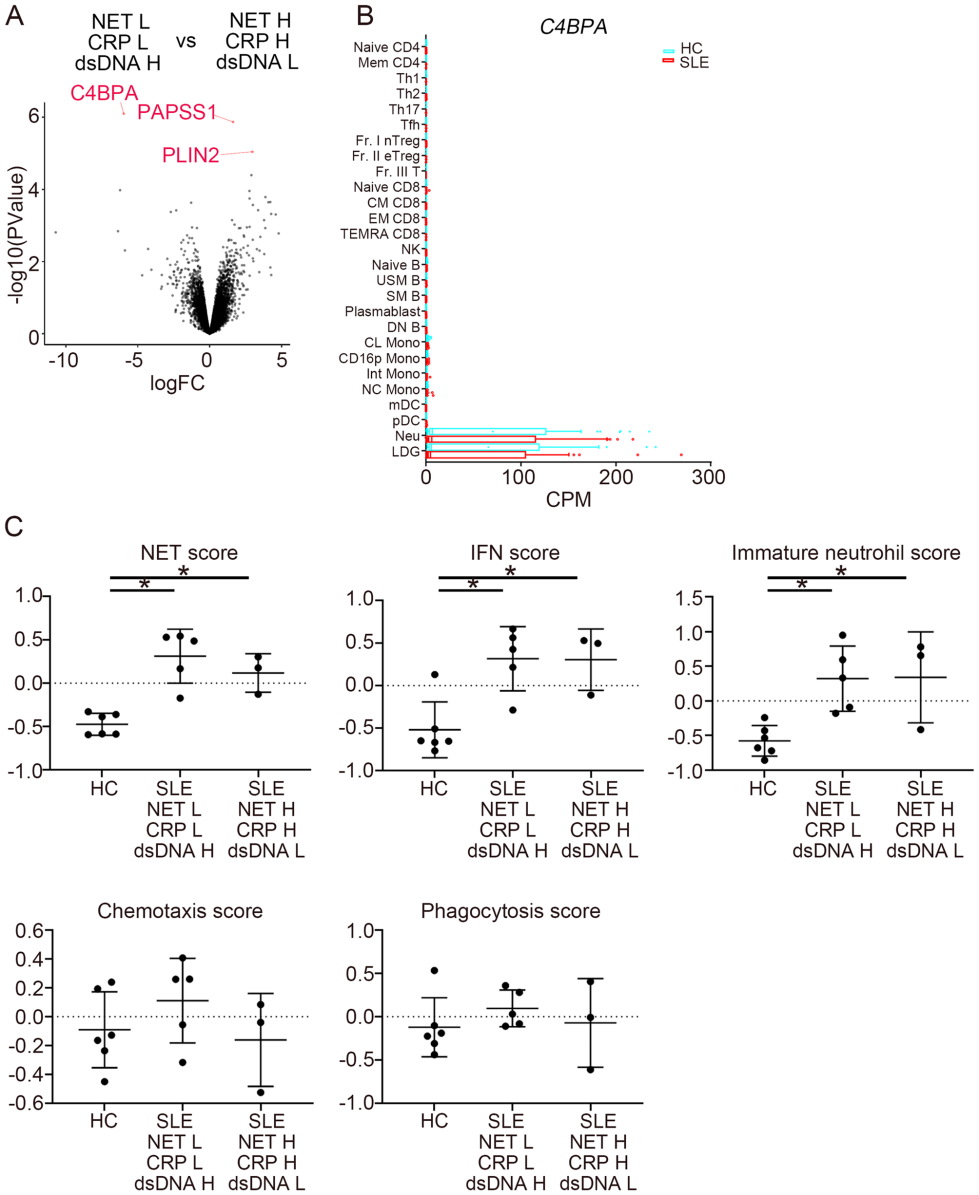
Transcriptomic analysis of neutrophils. (**A**) A volcano plot of differential gene expression of neutrophils. DsDNA represents anti-dsDNA antibody titer. mRNA expression between patients with low MPO-DNA complex/CRP and high anti-dsDNA antibody titers (n = 5), and high MPO-DNA complex/CRP and low anti-dsDNA antibody titers (n = 3) was compared. Upregulated or downregulated genes with FDR < 0.05 are in red. (**B**) mRNA expression of *C4BPA* in HCs and SLE patients from the ImmuNexUT database. (**C**) NET score, interferon score, immature neutrophils score, chemotaxis score, and phagocytosis score of HCs (n = 6) and lupus patients (n = 5 for patients with low MPO-DNA complex/CRP and high anti-dsDNA antibody titers and n = 3 for patients with high MPO-DNA complex/CRP and low anti-dsDNA antibody titers) were calculated by the GSVA using previously reported gene sets. DsDNA represents anti-dsDNA antibody titer. NET associated genes consist of *CAMP*, *PRTN3*, *HP1BP3*, *GSDMD*, *CASP4*, *SELPLG*, *RAC2*, *MLKL*, *PADI4*, *S100A8*, *S100A9*, *MMP9*, *ITGB2*, *PADI2*, *LCN2*, *MMP8*, *RAGE*, and *CORO1A* from the previous report by Vanderbeke et al.^19^. *P < 0.05; one-way ANOVA with Tukey’s multiple comparisons test. Error bars represent mean ± SD. Anti-dsDNA: anti-double stranded DNA antibody; CD16p Mono: CD16-positive monocytes; CL Mono: classical monocytes; CM DC8: central memory CD8 T cells; CPM: counts per million; DN B: double negative B cells; EM CD8: effector memory CD8 T cells; Fr. I nTreg: fraction I naïve regulatory T cells; Fr. II eTreg: fraction II effector regulatory T cells; Fr. III T: fraction III non-regulatory T cells; GSVA: Gene Set Variation Analysis; H: high; HC; healthy control; IFN: interferon; Int Mono: intermediate monocytes; L: low; LDG: low-density granulocytes; mDC: myeloid dendritic cells; Mem CD4: memory CD4 T cells; Naïve B: naïve B cells; Naïve CD4: naïve CD4 T cells; Naïve CD8: naïve CD8 T cells; NC Mono: non-classical monocytes; Neu: neutrophils; NET: neutrophil extracellular trap; NK: natural killer cells; pDC: plasmacytoid dendritic cells; SLE: systemic lupus erythematosus; SM: switched memory B cells; TEMRA CD8: CD8^+^ T effector memory CD45RA^+^ cells; Tfh: T follicular helper cells; Th1: T helper 1 cells; Th2: T helper 2 cells; Th17: T helper 17 cells; USM B: unswitched memory B cells.

To evaluate the transcriptomic characteristics of SLE patients with high levels of serum MPO-DNA complex and CRP, we compared neutrophil transcriptomes of SLE patients with high and low levels of MPO-DNA complex and CRP levels, and high and low anti-dsDNA antibody titers. We analyzed patients in two groups. One group consisted of patients with low MPO-DNA complex and low CRP with high anti-dsDNA antibody titers and the other group consisted of patients with high MPO-DNA complex and high CRP with low anti-dsDNA antibody titers. Transcriptomic data came from the other cohort (ImmuNexUT database) (Supplementary Table S5). DEGs of neutrophils in SLE patients in these groups are shown in Fig. 2A. Among the DEGs, *C4BPA* expression was increased in patients with low MPO-DNA complex and low CRP with high anti-dsDNA antibody titers, whereas *PAPSS1* and *PLIN2* were increased in patients with high MPO-DNA complex and high CRP with low anti-dsDNA antibody titers. According to the ImmuNexUT database, *C4BPA* was exclusively expressed in neutrophils and low-density granulocytes (LDG) both in HCs and SLE patients (Fig. 2B).

Functional signature gene scores were analyzed in neutrophils (Fig. 2C). NET score was calculated by adopting 18 NET formation associated genes used in a previous report^19^. We assessed how these gene signatures change by calculating NET score based on the up- or downregulation of their expression. The 18 genes consist of *CAMP*, *PRTN3*, *HP1BP3*, *GSDMD*, *CASP4*, *SELPLG*, *RAC2*, *MLKL*, *PADI4*, *S100A8*, *S100A9*, *MMP9*, *ITGB2*, *PADI2*, *LCN2*, *MMP8*, *RAGE*, and *CORO1A*. These gene set contains genes essential for NET production (*PADI4*) and gene encode intracellular protein in neutrophils (*S100A8*, *S100A9*). Interestingly, there was no difference in NET-associated gene scores, IFN scores, and immature neutrophil scores regardless of serum NET levels (Fig. 2C). Other scores related to other neutrophil functions, such as chemotaxis and phagocytosis, were almost identical between HCs and lupus patients (Fig. 2C). Therefore, we observed a dissociation between serum MPO-DNA complex levels and NET-associated gene signatures in neutrophils. We next evaluated NET degradation activity, as this impaired in lupus patients due to the presence of DNase1 inhibitors or anti-NET antibodies^23^. NET degradation was almost identical across serum samples from all groups (Supplementary Fig. S2). Therefore, low serum MPO-DNA complex levels were closely associated with high anti-dsDNA titers, but neutrophil activation did not differ between serum MPO-DNA complex high and low patients.

### Association between serum cytokine and functional protein levels and MPO-DNA complex levels

**Figure 3:**
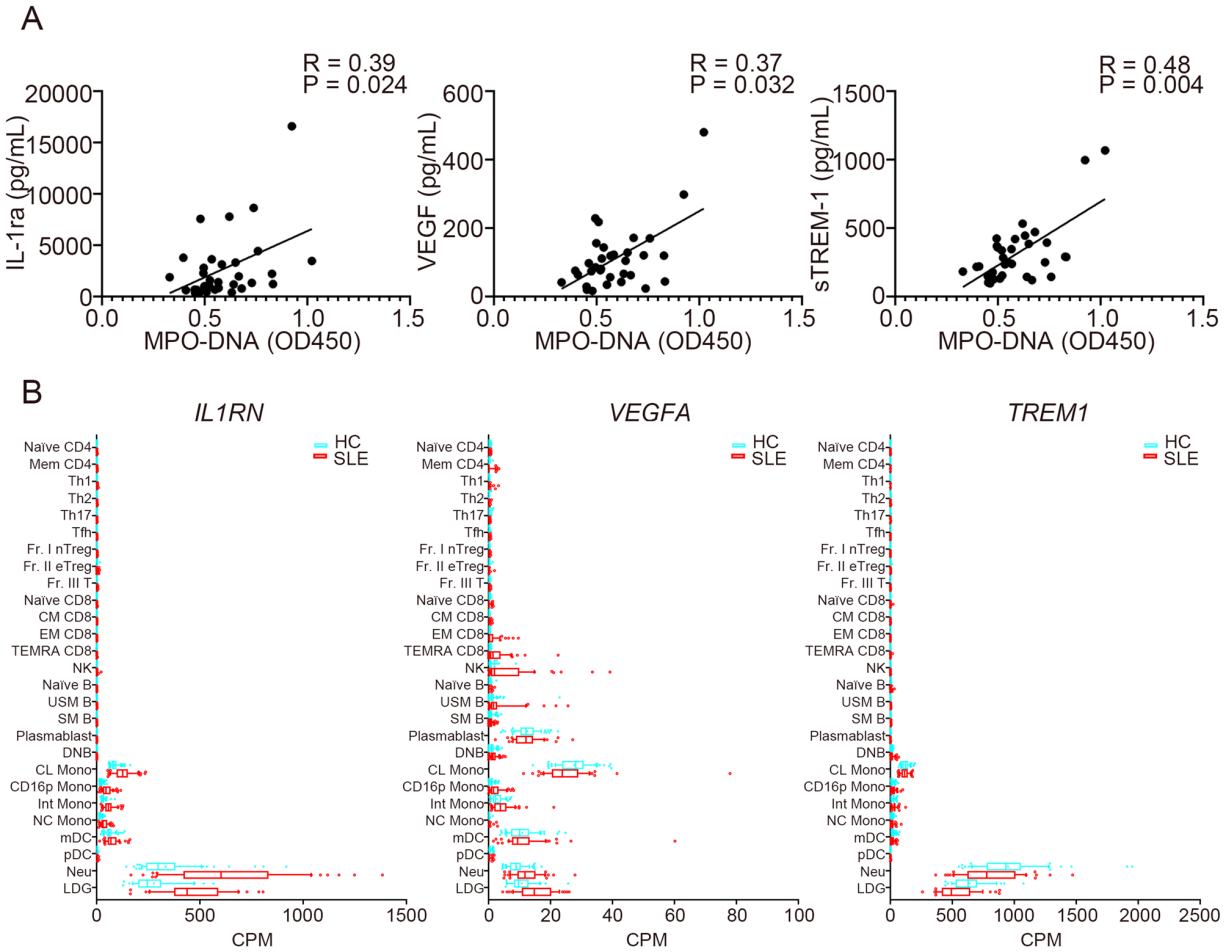
Serum MPO-DNA complex levels and correlations with cytokine and functional protein levels. (**A**) Correlations between serum MPO-DNA complex levels and serum IL-1RA, VEGF, and sTREM1 levels were analyzed (n = 33 for each; Spearman’s rank correlation coefficients). (**B**) mRNA expression of *IL1RN*, *VEGF*, and *TREM1* in HCs and SLE patients from the ImmuNexUT database. CD16p Mono: CD16-positive monocytes; CL Mono: classical monocytes; CM DC8: central memory CD8 T cells; CPM: counts per million; DN B: double negative B cells; EM CD8: effector memory CD8 T cells; Fr. I nTreg: fraction I naïve regulatory T cells; Fr. II eTreg: fraction II effector regulatory T cells; Fr. III T: fraction III non-regulatory T cells; HC: healthy controls; Int Mono: intermediate monocytes; LDG: low-density granulocytes; mDC: myeloid dendritic cells; Mem CD4: memory CD4 T cells; Naïve B: naïve B cells; Naïve CD4: naïve CD4 T cells; Naïve CD8: naïve CD8 T cells; NC Mono: non-classical monocytes; Neu: neutrophils; NK: natural killer cells; pDC: plasmacytoid dendritic cells; SLE: systemic lupus erythematosus; SM: switched memory B cells; sTREM-1: soluble TREM-1; TEMRA CD8: CD8^+^ T effector memory CD45RA^+^ cells; Tfh: T follicular helper cells; Th1: T helper 1 cells; Th2: T helper 2 cells; Th17: T helper 17 cells; USM B: unswitched memory B cells.

We next analyzed the association between serum cytokines and functional protein levels and MPO-DNA complex levels in SLE patients (Supplementary Table S6). Among the measured proteins, interleukin (IL)-1RA, vascular endothelial growth factor (VEGF), and soluble TREM-1 (sTREM-1) levels positively correlated with serum levels of the MPO-DNA complex (Fig. 3A, Supplementary Table S6). Statistical significance was still observed in sTREM-1 when corrected for multiple comparisons. The ImmuNexUT database showed that main cell types that expressed *IL1RN* and *TREM1* were neutrophils and LDG both in HCs and SLE patients, while monocytes, as well as neutrophils and LDG, expressed *VEGFA* (Fig. 3B). Additionally, serum interferon (IFN)-alpha levels were higher in anti-dsDNA antibody-positive patients (Supplementary Table S7).

### Cluster analysis of SLE patients

Based on these observations, we performed cluster analysis on a cohort of SLE patients in order to characterize serum MPO-DNA high patients in relation to laboratory tests commonly performed in the clinical setting (Table 2). We selected the variables for cluster analysis based on their correlation with MPO-DNA complex. The clinical parameters of anti-dsDNA titers, CRP levels, and monocyte count were selected, because there was a moderate positive correlation between these parameters and serum MPO-DNA complex levels. Additionally, serum sTREM-1 level positively correlated with MPO-DNA complex with the highest correlation coefficient and statistical significance remained after correction for multiple comparisons (Supplementary Table S6). For these reasons, we chose MPO-DNA complex, anti-dsDNA antibody titer, CRP, monocyte, and sTREM-1 as parameters for the cluster analysis. Patients were divided into three clusters: (1) low MPO-DNA complex/CRP/monocyte/sTREM-1 and high anti-dsDNA antibody titer, (2) the intermediate cluster, and (3) high MPO-DNA complex/CRP/monocyte/sTREM-1 and low anti-dsDNA antibody titer (Table 2, Supplementary Fig. S3).

**Table 2:** Clustering of SLE patients.

Cluster	N	MPO-DNA (mean [SD])	Anti-dsDNA Ab (mean [SD])	CRP (mean [SD])	Mono (mean [SD])	sTREM-1 (mean [SD])
1	4	0.47 (0.11)	321.68 (75.62)	0.23 (0.18)	228.00 (94.88)	281.04 (166.9)
2	26	0.57 (0.11)	25.00 (33.31)	1.43 (2.64)	254.27 (179.0)	264.84 (120.6)
3	3	0.92 (0.097)	3.17 (1.36)	12.87 (6.64)	866.67 (416.3)	785.59 (429.4)

Patients were grouped using K-means clustering. Data are presented as means.

*Anti-dsDNA Ab* anti-double stranded DNA antibody, *CRP* C-reactive protein, *Mono* monocyte, *MPO-DNA* myeloperoxidase-DNA, *SLE* systemic lupus erythematosus, *SD* standard deviation, *sTREM-1* soluble TREM-1.

**Figure 4:**
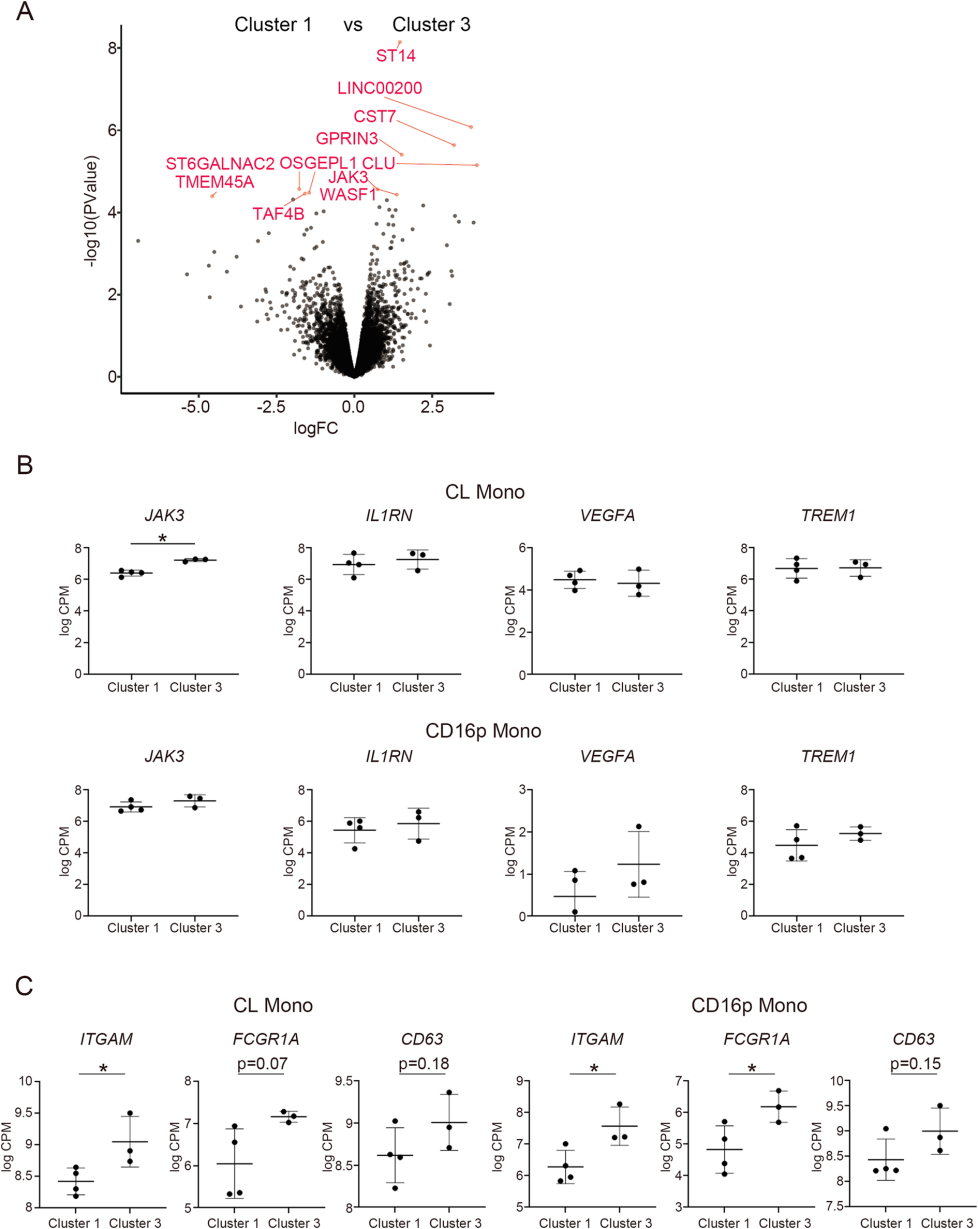
Transcriptomic analysis of monocytes. (**A**) A volcano plot of differential gene expression of classical monocytes. We defined cluster 1 as patients with low serum MPO-DNA complex and CRP, and positive for anti-dsDNA antibody (n = 4), and cluster 3 as patients with high serum MPO-DNA complex and CRP, and negative for anti-dsDNA antibody (n = 3). Upregulated or downregulated genes with FDR < 0.05 are in red. (**B**) *JAK3*, *IL1RN*, *VEGF*, and *TREM1* expression of classical monocytes (top) and CD16 positive monocytes (bottom) was compared between cluster 1 and cluster 3. (**C**) *ITGAM*, *FCGR1A*, and *CD63* expression of classical monocytes (left) and CD16 positive monocytes (right) was compared between cluster 1 and cluster 3 in the ImmuNexUT database. *P < 0.05; unpaired t-test. Error bars represent mean ± SD. Anti-dsDNA: anti-double stranded DNA antibody; CD16p Mono: CD16-positive monocytes; CL Mono: classical monocytes; CPM: counts per million; CRP; C-reactive protein; FDR: false discovery rate; MPO: myeloperoxidase.

To evaluate for a possible association between enhanced NET formation and monocyte activation with CRP elevation, we chose the patient data which correspond to cluster 1 and cluster 3 from the other cohort (ImmuNexUT database). We then compared monocyte transcriptomes from SLE patients in cluster 1 and cluster 3 from the ImmuNexUT database (Supplementary Table S8), to examine the hypothesis that there would be an association between high serum MPO-DNA complex and monocyte activation. DEGs of classical monocytes between SLE patients in clusters 1 and 3 are shown in Fig. 4A. Among the DEGs, expression level of *JAK3*, which encodes the Janus Kinase 3 (JAK3) protein, was increased in cluster 3. JAK3 is one of the intracellular protein tyrosine kinases that mediates inflammation^24^. In contrast to classical monocytes, DEGs were not detected in CD16-positive monocytes (data not shown). The difference in the mRNA expression levels of *IL1RN*, *VEGFA*, and *TREM1* was not observed either in classical monocytes or CD16-positive monocytes (Fig. 4B). In addition, mRNA expression of the monocyte activation marker *ITGAM* was significantly upregulated both in classical- and CD16-positive monocytes of the patients in cluster 3 compared with the patients in cluster 1 (Fig. 4C). Other activation markers *FCGR1A* and *CD63* were significantly or tended to be upregulated in patients in cluster 3 compared to patients in cluster 1 (Fig. 4C). *FCGR1A* tended to be upregulated in classical monocytes and significantly upregulated in CD16-positive monocytes while *CD63* tended to be upregulated in both classical monocytes and CD16-positive monocytes (Fig. 4C). Furthermore, ImmuNexUT database indicated that mRNA expression of *ITGAM* , *FCGR1A*, and *CD63* was upregulated both in classical- and CD16 positive- monocytes of lupus patients compared with HCs (Supplementary Fig. S4). These results suggested that there is increased monocyte activation in lupus patients with high level of serum NETs.

### Monocyte stimulation with NETs or a NET-containing immune complex

**Figure 5:**
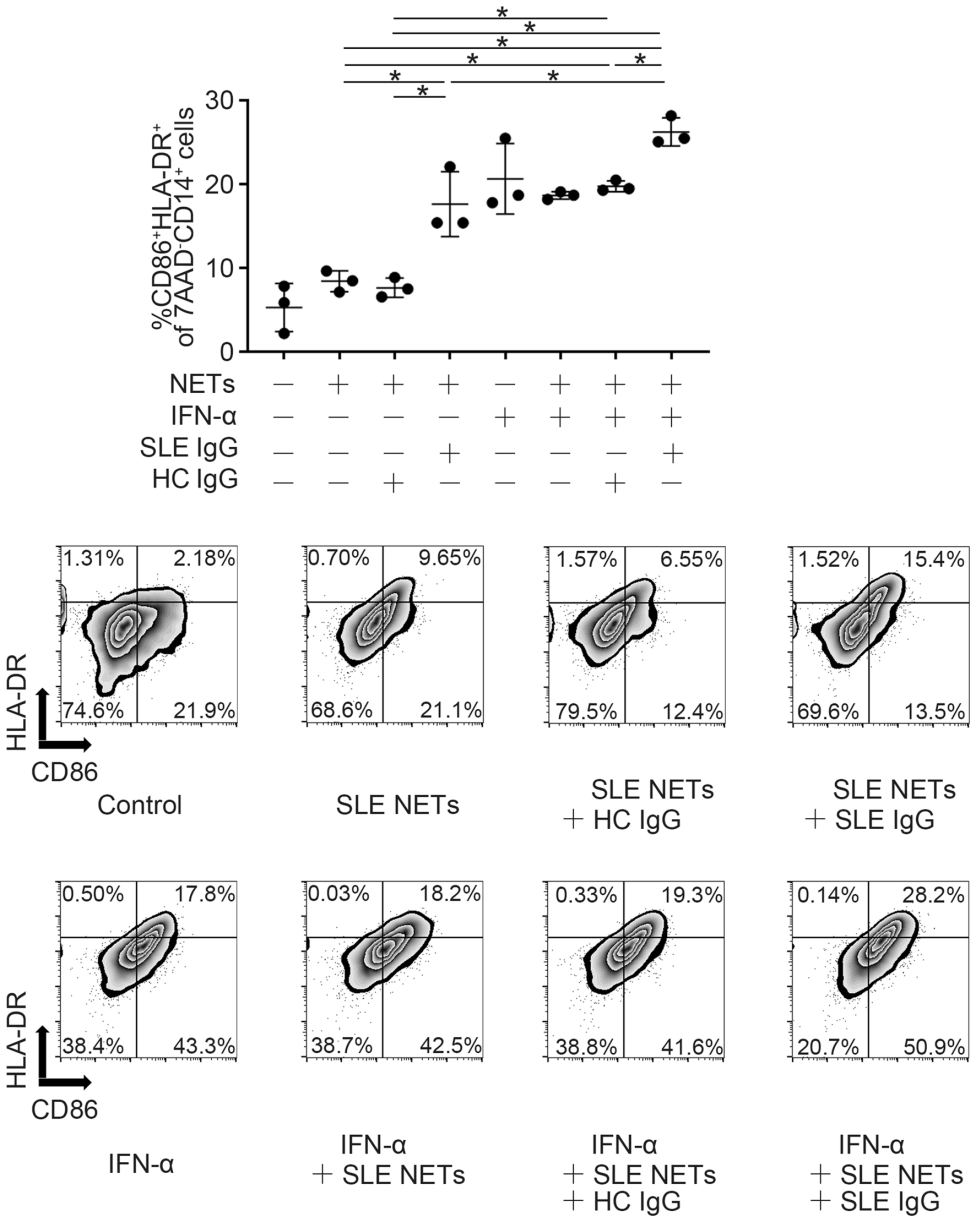
Monocyte stimulation with immune complexes. (Top) Monocytes were seeded at 1 × 10^6^ cells/mL and stimulated with 300 ng/mL of SLE patient–derived NETs or with an immune complex (SLE NETs-SLE IgG [10 µg/mL] or SLE NETs-HC IgG [10 µg/mL]), with or without IFN-α, for 2 days. Monocyte activation was evaluated by the proportion of activated monocytes (7AAD^-^CD14^+^CD86^+^HLA-DR^+^) using a flow cytometer. n = 3 for each stimulation condition. (Bottom) Representative figures of flow cytometric analysis from the conditions on the top. *P < 0.05; one-way ANOVA with Tukey’s multiple comparisons test. Error bars represent mean ± SD (Control, NETs, NETs + HC/SLE IgG, and IFN-α + NETs + HC/SLE IgG were used for multiple comparisons, and error bars were not shown for the result of the comparison between control and each condition.). HC: healthy control; IFN-α: interferon alpha; IgG: immunoglobulin G; NETs: neutrophil extracellular traps; SLE: systemic lupus erythematosus.

Finally, in order to examine the association between the enhancement of NET formations and monocyte activation in vitro, we assessed monocyte activation following stimulation with NETs or a NET-containing immune complex. When classical monocytes were stimulated with NETs alone, monocyte activation was minimal, although there was a slight shift to their activation assessed by flow cytometry (Fig. 5, Supplementary Fig. S5). However, when stimulated with an immune complex composed of NETs and IgG from SLE patients, monocytes became activated, and the activation was enhanced when the monocytes were primed with IFN-α (Fig. 5, Supplementary Fig. S5). In contrast, monocyte activation did not occur after stimulation with immune complexes composed of NETs with IgG from HCs.

## Discussion

SLE is a genetically and phenotypically heterogeneous disease. To achieve precision medicine for patients with SLE, defining SLE subpopulations based on differing immunological backgrounds is necessary. Indeed, SLE subpopulations have been categorized by immunophenotyping peripheral blood immune cells through heterogeneity in the helper T cells^25^. Transcriptional analysis has also been used to identify characteristics of active SLE. Notably, a neutrophil activation signature was associated with active lupus nephritis, which along with other studies has led to the thought that neutrophils play a crucial role in the pathogenesis of SLE^26^. Neutrophils can cause end organ damage and NETosis can lead to thrombosis and endothelial damage^27^. Mistry et al. demonstrated that the type I IFN gene expression signature was elevated in LDGs, by single-cell RNA sequencing of peripheral blood mononuclear cells from SLE patients^20^. In addition, intermediate-matured CD10^+^ LDGs showed proinflammatory phenotypes and had a strong association with organ damage^20^. Here, our study demonstrated that a subpopulation of SLE patients could be classified by serum levels of the MPO-DNA complex, CRP, and anti-dsDNA antibodies. These three serum markers could represent neutrophil activation (MPO-DNA complex), systemic inflammation (CRP), and B-cell responses (anti-dsDNA antibodies). Combination of surrogate markers of these different immunological processes could classify clinical subpopulations of SLE patients, and this approach might be useful for establishing personalized therapeutic targets.

Interestingly, a dissociation between serum MPO-DNA complex levels and the NET gene score in the neutrophil transcriptome was demonstrated. Our study found that serum MPO-DNA complex levels were relatively low in anti-dsDNA antibody-positive SLE patients. This indicates that serum NET elevation could be caused by the absence of serum anti-dsDNA antibodies, rather than by neutrophil activation. In the subpopulation of patients characterized by low serum MPO-DNA complex levels, immune complex formation was increased, and we hypothesize that NET-anti-dsDNA antibody immune complexes facilitates their binding to Fc gamma receptor-expressing tissue in patients with high level of anti-dsDNA antibody. As a consequence, serum level of NET remnants would be low in these patients even though NET degradation is identical between the high and low MPO-DNA complex subgroups. As our in vitro experiment indicated that immune complex activated monocytes, small amounts of immune complexes, which did not deposit to the tissue and retained in the peripheral blood of serum MPO-DNA high patients, could stimulate monocytes. Further study will be necessary to determine the difference and clinical significance between circulating and tissue deposited NET immune complexes.

As NETs are a source of autoantigen, a deficiency in their degradation could lead to more efficient antigen presentation. Thus, a higher amount of circulating NETs could promote a higher amount of anti-dsDNA antibody production. Indeed, Hakkim et al. showed in the German cohort that NET degradation activity was impaired in a subset of lupus patients because of the presence of DNase1 inhibitors or anti-NET antibodies, and NET non-degraders had significantly higher anti-dsDNA antibody titers than NET degraders^23^. In addition, Bruschi et al. demonstrated the positive correlation between anti-dsDNA antibody titer and levels of serum NETs in patients with incident SLE and lupus nephritis in the Italian cohort^28^. These are in contrast with our findings that patients with higher levels of serum NETs and normal degradation capacity are characterized by low levels of serum anti-dsDNA antibodies. We speculate that these conflicting data would be mainly due to patient characteristics of the cohorts. These German and Italian cohorts mainly focused on the patient with lupus nephritis which may differ from our “inflammatory” lupus population. Considering our cohort consisted of the patients with high disease activity (mean SLEDAI-2K of 12.36), and the frequency of active nephritis was relatively low (18.18%), the frequency of inflammatory lupus patients characterized by CRP elevation could be higher in our cohort in comparison to these other cohorts. Furthermore, the inclusion of healthy controls might have enabled the Italian cohort to detect positive correlation between circulating NET levels and anti-dsDNA antibody titers.

Notably, in our study, *C4BPA* was identified as a DEG in neutrophils of patients with low levels of serum MPO-DNA complex. C4b-binding protein (C4BP) is a protein synthesized mainly in the liver and inhibits the classical and lectin pathways of complement activation^29^. C4BP also inhibits phagocytosis of apoptotic cells^30^. We hypothesize that enhanced *C4BPA* expression in neutrophils inhibits phagocytosis of apoptotic cells, which can lead to an increase in serum autoantigens and consequent anti-dsDNA antibody development in SLE patients. In addition, *C4BPA* was reported as a risk gene of venous thromboembolism with expression quantitative trait loci effects^31^. The precise roles of *C4BPA* in neutrophils in the context of SLE pathogenesis will need to be examined in the future.

Previous report indicated that CRP-lowering polymorphism rs1205 could associate the low CRP levels in lupus patients^32^. As such, CRP level is generally low in lupus patients and it is not a typical marker involved in SLE. However, some lupus patients present with a proinflammatory phenotype, with elevated serum CRP and higher frequencies of pleuritis and myositis^3,4^. Interestingly, in the subpopulation of patients with high serum MPO-DNA and negative anti-dsDNA antibody titers, inflammatory phenotypes were observed. This subpopulation was characterized by a proinflammatory phenotype, including an elevation of serum CRP and higher frequencies of pleuritis and myositis, which is consistent with previous reports^3,4^. Casey et al. demonstrated the correlation between circulating NET complex levels and type I IFN pathway activity^33^, while the correlation between circulating MPO-DNA levels and inflammatory cytokine protein levels including IFN-α was not striking. We demonstrated serum levels of IL-1RA, VEGF, and sTREM-1 positively correlated with serum levels of the MPO-DNA complex, and the ImmuNexUT database revealed that mRNA expression levels of these functional proteins were enhanced mainly in myeloid cells, including neutrophils and monocytes. Considering that the transcriptomic profile was almost identical in patients with high or low serum MPO-DNA complex levels, the difference in serum protein expression may be derived from the greater number of neutrophils, rather than from a difference in neutrophil function per se. While NETosis is induced by various stimuli including IFN-α, our data might indicate that TREM-1 potentiates NET release in lupus patients with high serum MPO-DNA levels as observed in sepsis^34^. Therefore, it is possible that sTREM-1 might be released during NET formation and also contribute to amplify it in lupus pathology. Although the exact function of sTREM-1 in NETs is still unclear, further study is warranted to confirm if sTREM-1 production is increased in NET formation, and that NET formation is enhanced following neutrophil stimulation with sTREM-1 in lupus pathology.

In our study, elevated monocyte count and CRP levels were noted in SLE patients with high MPO-DNA complex levels. Also, classical monocytes were activated following application of an immune complex composed of NETs and IgG from SLE patients. Although proinflammatory response of monocytes was minimal when stimulated by NETs, monocyte-derived macrophages may have a more proinflammatory response with NET stimulation as shown in several reports^35,36^. Recent reports indicated that enhanced NETosis^18^ and classical monocytes drove a hyperinflammatory phenotype in COVID-19 patients^19^. In addition, SLE patients with high MPO-DNA complex levels had an inflammatory classical monocyte phenotype which was associated with *JAK3* expression. A recent report indicated that tofacitinib, a JAK inhibitor that mainly inhibits JAK3, reduced circulating NETs in lupus patients^37^. Collectively, classical monocytes could be activated with NETs or a NET-containing immune complex in patients with an inflammatory phenotype of lupus and COVID-19. Considering that serum MPO-DNA complex levels were decreased in lupus patients with a high titer of anti-dsDNA antibody, it is also possible that anti-dsDNA antibodies physiologically deplete circulating NETs and thus do not allow activation of macrophages by NET per se. We believe that a further detailed characterization of the proinflammatory subpopulation of lupus patients is required, including an analysis of the patient’s genetic background, to bolster the use of personalized medicine in the future. Anti-cytokine therapies might be appropriate for treating inflammatory states in subpopulations of patients with high levels of serum NETs.

The detailed mechanism of monocyte activation needs to be clarified. We hypothesize that the stimulatory effect of anti-dsDNA IgG antibody itself is weaker than that of immune complex based on the previous report demonstrating that IFN- α production by plasmacytoid dendritic cell was lower when NET-derived candidate peptides were depleted from immune complexes^7^. Based on previous reports, candidate signaling pathways for monocyte activation could be TLR7^38^, TLR9^7,39^ or cGAS-STING pathway^8^. ImmuNexUT database indicates *TLR9* mRNA expression in monocyte is much lower compared with pDCs, and *TLR7* and STING-encoding gene *TMEM173* expressed higher especially in classical monocytes (data not shown). Based on these data, TLR7 or cGAS-STING pathway is the most likely mechanism of monocyte activation with immune complexes although further study is warranted.

Monocytes are divided into three subsets, namely classical, intermediate and non-classical subsets^40^. Although we have not stimulated non-classical monocytes, which are considered pro-inflammatory ones, these monocytes might be activated as well following NET containing immune complex stimulation considering monocyte activation marker genes were upregulated both in classical- and CD16-positive monocytes in patients with high MPO-DNA, and high CRP with low anti-dsDNA antibody. As *TLR7* and STING-encoding gene *TMEM173* expression is higher in classical monocytes compared with CD16 positive- monocytes in the ImmunNexUT data base (data not shown), classical monocytes might be more prone to be activated with NET-containing immune complexes compared with CD16 positive- monocytes. The differential effect of NET containing immune complex on classical- and CD16 positive- monocytes should be clarified in future study.

We recently published a letter regarding the correlation between serum MPO-DNA complex and clinical parameters in giant cell arteritis or Takayasu arteritis^41^. In the correspondence, serum NET levels positively correlated with serum ferritin levels but did not correlate with white blood cell count, neutrophil count, monocyte count, and CRP levels in peripheral blood which is in contrast to what we observed in SLE patients. These results might indicate that serum NET levels are not just a marker of inflammation in general, although further validation is warranted in larger cohort including the patients with other immune-mediated diseases. We cannot completely eliminate the possibility that negative association between anti-dsDNA antibodies and NET remnants could be partially due to technical interference with anti-dsDNA antibodies in the patient sera. Further study is necessary to clarify this point in the larger cohorts.

There are a couple of limitations in this study worth noting. First, the study included limited numbers of samples. Moore et al. reported NETs levels were associated with arterial events and endothelial cell activation in a larger lupus cohort with patients who had lower disease activities (median SLEDAI-2K of 2)^42^ compared with our cohort (mean SLEDAI-2K of 12.36). They also showed levels of NETs did not correlate with SLEDAI-2K, which was consistent with our study. Although the patient background of our small lupus cohort could have biased the study results, as it primarily comprised hospitalized patients with high disease activity, we believe that this may have contributed to the identification of the novel inflammatory lupus subpopulation. In fact, we could see at least 2 distinct clusters (cluster 1 and cluster 3), and these clusters might represent 2 distinct populations which may partly account for the heterogeneity in lupus patients. Second, we should keep in mind that serum MPO-DNA complexes do not always represent NETs because recent reports showed that METs also contained MPO^13^. Hence, METs, as well as NETs, might have partially contributed to our results. Another report indicates the ELISA detection of MPO-DNA complexes in human plasma sample tends to be error-prone because of their low specificity^43^. Additionally, serum NET levels do not necessarily reflect physiologic levels of NETs, as artificial NETs are formed upon serum processing due to coagulation^44^. Therefore, comparing additional NET markers and confirming the correlation between plasma NET remnant levels and the levels of NET structures from isolated lupus neutrophils are warranted. Third, we should also be mindful of the fact that the correlation between transcriptome signatures and NET release in lupus pathology is still unclear. Khan et al. demonstrated inhibitors of transcription suppressed NETosis^45^, while the suppression was not observed in other report by Sollberger et al.^46^. Considering that NET components are not completely identical even between rheumatoid arthritis and SLE^47^, further study is necessary to detect lupus-specific NET formation associated genes as well as their functional importance.

In conclusion, we identified SLE subpopulations based on serum NET levels; SLE patients with high serum NET were characterized by low serum anti-dsDNA antibody titers and high inflammatory responses with high frequencies of pleuritis and myositis, whereas patients with low serum NET levels were characterized by higher anti-dsDNA antibody titers and immune complex formation. Importantly, transcriptome analysis revealed that NET gene scores were equivalently increased both in the high and low serum NET subpopulations. Further characterization of this SLE subpopulation with high serum NET may allow for a more individualized approach to treatment with the potential to target proinflammatory processes (such as IL-1 inhibitor or IL-6 inhibitor) rather than antibody-dependent processes (such as CD20-specific antibody or antibody against B-cell activating factor) in this group of patients. Thus, serum MPO-DNA complex-based clustering of SLE subpopulations might work for supporting precision medicine in SLE.

## Supplementary Information

Supplementary Information 1.

Supplementary Information 2.

Supplementary Information 3.

Supplementary Information 4.

Supplementary Information 5.

Supplementary Information 6.

## Data Availability

The datasets analyzed during the current study are available from the corresponding author on reasonable request.
