# Supplementary material for: Serum extracellular traps associate with the activation of myeloid cells in SLE patients with the low level of anti-DNA antibodies
Source: Sci Rep. 2022 Nov 1;12:18397. doi: 10.1038/s41598-022-23076-1 (PMC9626644; doi:10.1038/s41598-022-23076-1)
Supplement: Supplementary file 1 — Supplementary Information 1. [file 41598_2022_23076_MOESM1_ESM.docx]

Supplementary Materials

# Supplementary Methods

**Neutrophil extracellular trap (NET) degradation assay**

NET degradation was assessed as previously reported, with minor modifications [1]. In brief, neutrophils from healthy donors were isolated using Polymorphprep (Axis-Shield) and were resuspended with RPMI (Gibco). Neutrophils were seeded with 1 × 10^5^ cells into each well of a 96-well black plate (PerkinElmer) coated with 0.001% poly-L-lysine (Sigma). Neutrophils were stimulated with 20 nM PMA (Sigma) for 4 hours at 37 °C, 5% CO_2_. Then, the media was removed and washed with phosphate-buffered saline. NETosis was quantified by incubating the cells for 30 minutes at 37 °C, 5% CO_2_ with 1 µM of SYTOX Green (Thermo Fisher) and fluorescence was quantified at excitation and emission wavelengths of 504 nm and 523 nm, respectively, using a 2300 EnSpire (PerkinElmer). For NET degradation assessment, NETs were incubated for 90 minutes at 37 °C, 5% CO_2_ with human serum diluted to 5% in nuclease buffer (10 mM Tris-HCl pH 7.5, 10 mM MgCl_2_, 2 mM CaCl_2_, and 50 mM NaCl). Then, fluorescence was requantified at excitation and emission wavelengths of 504 nm and 523 nm, respectively, using a 2300 EnSpire (PerkinElmer).

**Reference**

1 Zuo, Y. *et al.* Anti-Neutrophil Extracellular Trap Antibodies and Impaired Neutrophil Extracellular Trap Degradation in Antiphospholipid Syndrome. *Arthritis Rheumatol* **72**, 2130-2135, doi:10.1002/art.41460 (2020).

# Supplementary figure legends

**Supplementary Figure S1. Comparison of clinical features between patients with anti-dsDNA antibody-positive or -negative status.**
SLEDAI-2K (A), and other clinical parameters, such as lymphocyte and monocyte counts, and CH50 and C3 levels (B) were compared between anti-dsDNA antibody-positive (n = 15) and -negative (n = 18) patients. *P < 0.05; Mann Whitney U test. Error bars represent median ± IQR.
Anti-dsDNA: anti-double stranded DNA antibody; Lym: lymphocyte; Mono: monocyte; SLEDAI-2K: systemic lupus erythematosus disease activity index 2000.

**Supplementary Figure S2. Percentage of neutrophil extracellular trap (NET) degradation in healthy controls and systemic lupus erythematosus patients.**
Percentage of NET degradation in HCs (n = 6) and patients with low serum MPO-DNA complex and CRP, and positive for anti-dsDNA antibody (n = 5), and with high serum MPO-DNA complex and CRP, and negative for anti-dsDNA antibody (n = 3) was assessed.
Anti-dsDNA: anti-double stranded DNA antibody; CRP: C-reactive protein; H: high; HCs: healthy controls; L: low; NETs: neutrophil extracellular traps.

**Supplementary Figure S3. Serum myeloperoxidase (MPO)-DNA complex levels in patients of each cluster.**
Serum MPO-DNA complex levels in cluster 1 (n = 4), cluster 2 (n = 26), and cluster 3 (n = 3) in systemic lupus erythematosus patients shown in Table 2. *P < 0.05; one-way ANOVA with Tukey’s multiple comparisons test. Error bars represent mean ± SD.

**Supplementary Figure S4. *ITGAM*, *FCGR1A*, and *CD63* expression of monocytes in HCs and SLE.**
(A) *ITGAM*, *FCGR1A*, and *CD63* expression of classical monocytes was compared between HCs and SLE in the ImmuNexUT database. (B) *ITGAM*, *FCGR1A*, and *CD63* expression of CD16 positive- monocytes was compared between HC and SLE in the ImmuNexUT database. ****P < 0.0001; unpaired t-test. Error bars represent mean ± SD.
HCs: healthy controls; SLE: systemic lupus erythematosus.

**Supplementary Figure S5. Monocyte stimulation with immune complexes.**
Monocytes were seeded at 1 × 10^6^ cells/mL and stimulated with 300 ng/mL of SLE patient–derived NETs or with an immune complex (SLE NETs-SLE IgG [10 µg/mL] or SLE NETs-HC IgG [10 µg/mL]), with or without IFN-α, for 2 days. Monocyte activation was evaluated by the proportion of activated monocytes (7AAD^-^CD14^+^CD86^+^HLA-DR^+^) using a flow cytometer. CD86 MFI (left) and HLA-DR MFI (right) of 7AAD^-^CD14^+^ cells are shown. n = 3 for each stimulation condition. *P < 0.05; one-way ANOVA with Tukey’s multiple comparisons test. Error bars represent mean ± SD (Control, NETs, NETs + HC/SLE IgG, and IFN-α + NETs + HC/SLE IgG were used for multiple comparisons, and error bars were not shown for the result of the comparison between control and each condition.).
HC: healthy control; IFN-α: interferon alpha; IgG: immunoglobulin G; MFI: median fluorescence intensity; NETs: neutrophil extracellular traps.

# Supplementary Tables

**Supplementary Table S1. Demographic and clinical features of patients with SLE.**

|  | **SLE (n = 33)** | **HC (n = 19)** | **P value** |  |
| --- | --- | --- | --- | --- |
| Age (years; mean [SD]) | 48.33 (14.87) | 50.16 (9.14) | 0.63 |  |
| Female (%) | 28/33 (84.85) | 17/19 (89.47) | 1.00 |  |
| Disease duration  (months; median [IQR]) | 11.00  (0.00 - 187.50) | - | - |  |
| SLEDAI-2K score (mean [SD]) | 12.36 (7.63) | - | - |  |
| Disease manifestation |  |  |  |  |
| Constitutional (%) | 10/33 (30.30) | - | - |  |
| Skin (%) | 10/33 (30.30) | - | - |  |
| Musculoskeletal (%) | 18/33 (54.55) | - | - |  |
| Renal (%)  Active nephritis (%) | 10/33 (30.30)  6/33 (18.18) | - | - |  |
| Neurological (%) | 8/33 (24.24) | - | - |  |
| Cardiopulmonary (%) | 5/33 (15.15) | - | - |  |
| Hematological (%) | 10/33 (30.30) | - | - |  |
| Laboratory index |  |  |  |  |
| Anti-dsDNA (%) | 15/33 (45.45) | - | - |  |
| Anti-RNP (%) | 19/33 (57.58) | - | - |  |
| Anti-Sm (%) | 14/33 (42.42) | - | - |  |
| Anti-SSA (%) | 25/33 (75.76) | - | - |  |
| Anti-SSB (%) | 8/31 (25.81) | - | - |  |
| Lupus anticoagulant (%) | 11/33 (33.33) | - | - |  |
| Anti-cardiolipin IgG (%) | 8/33 (24.24) | - | - |  |
| Anti-cardiolipin β2GPI (%) | 5/33 (15.15) | - | - |  |
| Direct Coombs test (%) | 24/33 (72.73) | - | - |  |
| PSL dosage (mg; median [IQR]) | 0.00 (0.00 - 7.75) | - | - |  |
| Medication |  |  |  |  |
| HCQ (%) | 6/33 (18.18) |  |  |  |
| PSL (%) | 16/33 (48.48) |  |  |  |
| MMF (%) | 4/33 (12.12) |  |  |  |
| AZA (%) | 1/33 (3.03) |  |  |  |
| Tac (%) | 2/33 (6.06) |  |  |  |
| CyA (%) | 1/33 (3.03) |  |  |  |

Differences between groups in age (two-tailed unpaired *t* test) and in the proportion of categorical variables (Fisher’s exact test) were analyzed.

Anti-dsDNA: anti-double stranded DNA antibody; AZA: azathioprine; β2GPI: β2-glycoprotein I; CyA: cyclosporin A; HC: healthy control; HCQ: hydroxychloroquine; IgG: immunoglobulin G; IQR: interquartile range; MMF: mycophenolate mofetil; PSL: prednisolone; RNP: ribonucleoprotein; SD: standard deviation; SLE: systemic lupus erythematosus; SLEDAI-2K: systemic lupus erythematosus disease activity index 2000; Sm: Smith; Tac: tacrolimus.

**Supplementary Table S2. Correlation between MPO-DNA complex levels and clinical parameters in patients with SLE.**

|  | **N** | **R** | **P value** | **FDR** |
| --- | --- | --- | --- | --- |
| WBC | 33 | 0.40 | 0.022 | 0.058 |
| Neutrophil | 33 | 0.39 | 0.023 | 0.058 |
| Lymphocyte | 33 | 0.18 | 0.31 | 0.39 |
| Monocyte | 33 | 0.42 | 0.014 | 0.053 |
| Hemoglobin | 33 | 0.083 | 0.64 | 0.69 |
| Platelet | 33 | 0.042 | 0.82 | 0.82 |
| CRP | 33 | 0.59 | < 0.001 | 0.002 |
| ESR | 33 | 0.23 | 0.20 | 0.30 |
| CH50 | 33 | 0.16 | 0.36 | 0.42 |
| C3 | 33 | 0.27 | 0.13 | 0.24 |
| C4 | 33 | 0.22 | 0.22 | 0.30 |
| Anti-dsDNA Ab | 33 | -0.46 | 0.007 | 0.036 |
| IC-C1q | 22 | -0.43 | 0.048 | 0.10 |
| SLEDAI-2K | 33 | -0.25 | 0.16 | 0.26 |

Correlations between MPO-DNA complex levels and each clinical parameter were analyzed (Spearman’s rank correlation coefficient with Benjamini-Hochberg correction for multiple comparisons).

Anti-dsDNA Ab: anti-double stranded DNA antibody; C: complement component; CRP: C-reactive protein; ESR: erythrocyte sedimentation rate; FDR: false discovery rate; IC-C1q: C1q-binding immune complexes; MPO-DNA: myeloperoxidase-DNA; SLEDAI-2K: systemic lupus erythematosus disease activity index 2000; WBC: white blood cell.

**Supplementary Table S3. Comparison of clinical features between patients with high and low MPO-DNA complex levels**.

|  | **MPO-DNA low**  **(n = 20)** | **MPO-DNA high**  **(n = 13)** | **P value** |
| --- | --- | --- | --- |
| Female (%) | 18/20 (90.00) | 10/13 (76.92) | 0.36 |
| Anti-SSA (%) | 17/20 (85.00) | 8/13 (61.54) | 0.21 |
| Anti-SSB (%) | 6/19 (31.58) | 2/12 (16.67) | 0.43 |
| Anti-RNP (%) | 12/20 (60.00) | 7/13 (53.85) | 1.00 |
| Anti-Sm (%) | 10/20 (50.00) | 4/13 (30.77) | 0.31 |
| Lupus anticoagulant (%) | 5/19 (26.32) | 6/13 (46.15) | 0.28 |
| Anti-cardiolipin IgG (%) | 6/20 (30.00) | 2/13 (15.38) | 0.43 |
| Anti-cardiolipin β2GPI (%) | 3/20 (15.00) | 2/13 (15.38) | 1.00 |
| Direct Coombs test (%) | 15/20 (75.00) | 9/12 (75.00) | 1.00 |

The MPO-DNA “high” group was defined as OD value > 0.62 (mean + 2SD of HC). Differences between groups in the proportion of categorical variables was analyzed by Fisher’s exact test.

β2GPI: β2-glycoprotein I; IgG: immunoglobulin G; MPO-DNA: myeloperoxidase-DNA; RNP: ribonucleoprotein; Sm: Smith.

**Supplementary Table S4. Comparison of clinical features between patients with and without an anti-dsDNA antibody increase.**

|  | **Anti-dsDNA Ab Negative (n = 18)** | **Anti-dsDNA Ab Positive (n = 15)** | **P value** |
| --- | --- | --- | --- |
| MPO-DNA complex  (OD; median [IQR]) | 0.6455  (0.5403 - 0.7766) | 0.4950  (0.4620 - 0.5345) | 0.002 |
| SLEDAI-2K score  (median [IQR]) | 8.00  (2.75 – 11.25) | 18.00  (12.00 – 21.00) | 0.002 |
| WBC  (count/µL; median [IQR]) | 4750  (3850 - 7025) | 5100  (2000 - 6300) | 0.35 |
| Neutrophil  (count/µL; median [IQR]) | 3500  (2300 - 5450) | 4100  (1200 - 5000) | 0.61 |
| Lymphocyte  (count/µL; median [IQR]) | 1050  (700 - 1325) | 600  (400 - 900) | 0.003 |
| Monocyte  (count/µL; median [IQR]) | 300  (200 - 500) | 200  (100-300) | 0.017 |
| CRP  (mg/dL; median [IQR]) | 1.13  (0.16 - 7.46) | 0.31  (0.040 - 0.41) | 0.098 |
| CH50  (/mL; median [IQR]) | 42.20  (20.55 - 53.58) | 17.30  (9.90 – 29.80) | 0.025 |
| C3  (mg/dL; median [IQR]) | 71.00  (55.75 – 87.75) | 43.00  (32.00 – 55.00) | 0.009 |
| C4  (mg/dL; median [IQR]) | 10.50  (4.50- 17.25) | 6.00  (2.00 – 12.00) | 0.074 |

Differences between patients with and without an anti-dsDNA antibody increase were analyzed by Mann-Whitney *U* tests.

Anti-dsDNA Ab: anti-double stranded DNA antibody; C: complement component; CH50: total complement measurement; CRP: C-reactive protein; IQR: interquartile range; MPO-DNA: myeloperoxidase-DNA; SLEDAI-2K: systemic lupus erythematosus disease activity index 2000; WBC: white blood cell.

**Supplementary Table S5. Demographic and clinical features of HC and patients with SLE from ImmuNexUT Database in Figure 2 and Supplementary Figure S2.**

|  | **SLE (n = 8)** | | **HC (n = 6)** |
| --- | --- | --- | --- |
|  | **NET Low**  **CRP Low**  **dsDNA High**  **(n = 5)** | **NET High**  **CRP High**  **dsDNA Low**  **(n = 3)** |  |
| Age (years; mean [SD]) | 37.20 (14.24) | 47.33 (18.61) | 54.33 (10.46) |
| Female (%) | 5/5 (100) | 2/3 (66.67) | 6/6 (100) |
| CRP (mg/dL; median [IQR]) | 0.09 (0.02 – 0.10) | 2.89 (1.67 – 6.25) | - |
| Anti-dsDNA Ab  (IU/mL: mean [SD]) | 183.7 (138.6) | 2.5 (0.5) | - |

The MPO-DNA “high” group was defined as OD value > 0.41 (mean + 2SD of HC).

Anti-dsDNA Ab: anti-double stranded DNA antibody; CRP: C-reactive protein; HC: healthy control; IQR: IQR: interquartile range.

**Supplementary Table S6. Correlations between serum MPO-DNA complex and cytokine and functional protein levels in patients with SLE.**

|  | **N** | **R** | **P value** | **FDR** |
| --- | --- | --- | --- | --- |
| IL-1ra | 33 | 0.39 | 0.024 | 0.14 |
| IFN-α | 26 | -0.14 | 0.49 | 0.70 |
| TNF-α | 31 | 0.27 | 0.14 | 0.35 |
| IL-6 | 33 | 0.31 | 0.078 | 0.26 |
| IL-6R | 33 | 0.058 | 0.75 | 0.83 |
| IL-12/23 p40 | 25 | -0.14 | 0.50 | 0.70 |
| IL-18 | 33 | 0.29 | 0.11 | 0.30 |
| CCL2 | 33 | -0.050 | 0.78 | 0.83 |
| CCL3 | 27 | -0.037 | 0.86 | 0.86 |
| CCL4 | 33 | 0.14 | 0.45 | 0.70 |
| CX3CL1 | 33 | 0.078 | 0.67 | 0.83 |
| M-CSF | 26 | 0.070 | 0.73 | 0.83 |
| G-CSF | 33 | 0.16 | 0.38 | 0.70 |
| VEGF | 33 | 0.37 | 0.032 | 0.14 |
| sTREM-1 | 33 | 0.48 | 0.004 | 0.037 |
| C5a | 33 | 0.21 | 0.23 | 0.50 |

Correlations between MPO-DNA complex level and each cytokine and functional protein level were analyzed (Spearman’s rank correlation coefficient with Benjamini-Hochberg correction for multiple comparisons).

C5a: complement component 5a; CCL: C–C motif chemokine ligand; CX3CL1: C-X3-C motif chemokine ligand 1; FDR: false discovery rate; G-CSF: granulocyte colony stimulating factor; IFN: interferon; IL: interleukin; M-CSF: macrophage colony stimulating factor; MPO-DNA: myeloperoxidase-DNA; SLE, systemic lupus erythematosus; sTREM-1: soluble TREM-1; TNF: tumor necrosis factor; VEGF: vascular endothelial growth factor.

**Supplementary Table S7. Comparison between patients with and without anti-dsDNA antibody increases in cytokines and functional proteins.**

|  | **Anti-dsDNA Ab Negative (n = 18)** | **Anti-dsDNA Ab Positive (n = 15)** | **P value** |
| --- | --- | --- | --- |
| IL-1ra | 1205.00  (691.60 - 3359.00) | 1888.00  (950.70 - 3644.00) | 0.53 |
| IFN-α | 2.21  (0.00 - 3.72) | 4.74  (2.10 - 11.16) | 0.026 |
| TNF-α (pg/mL) | 10.13  (4.47 - 26.58) | 10.73  (5.36 - 18.61) | 0.94 |
| IL-6 | 6.92  (3.16 - 27.40) | 6.640  (3.010 - 15.51) | 0.73 |
| IL-6R | 35066  (25907 - 40266) | 34921  (31675 - 40442) | 0.68 |
| IL-12/23 p40 | 52.20  (35.28 - 71.01) | 61.39  (0.00 - 235.20) | 0.67 |
| IL-18 | 234.00  (201.90 - 696.00) | 283.10  (174.60 - 392.40) | 0.90 |
| CCL2 | 389.70  (260.00 - 800.60) | 567.20  (349.10 - 937.50) | 0.34 |
| CCL3 | 178.10  (55.61 - 372.60) | 140.00  (79.16 - 233.10) | 0.52 |
| CCL4 | 210.40  (136.10 - 284.40) | 233.60  (198.40 - 428.30) | 0.43 |
| CX3CL1 | 1865  (1171 - 2918) | 2675  (1464 - 3322) | 0.36 |
| M-CSF | 153.30  (45.95 - 351.90) | 137.80  (70.13 - 502.70) | 0.96 |
| G-CSF | 99.44  (91.21 - 106.90) | 99.71  (93.25 - 146.60) | 0.78 |
| VEGF | 119.40  (58.15 - 170.60) | 77.29  (41.67 - 111.00) | 0.16 |
| sTREM-1 | 270.30  (147.50 - 452.70) | 236.20  (154.80 - 368.90) | 0.36 |
| C5a | 64691  (58123 - 92499) | 52052  (33342 - 93610) | 0.23 |

Differences between patients with and without anti-dsDNA antibody increases were analyzed by Mann-Whitney *U* test. Data are presented as median (IQR) in pg/mL.

Anti-dsDNA Ab: anti-double stranded DNA antibody; CCL: C–C motif chemokine ligand; CX3CL1: C-X3-C motif chemokine ligand 1; G-CSF: granulocyte colony stimulating factor; IFN: interferon; IL: interleukin; IQR: interquartile range; M-CSF: macrophage colony stimulating factor; sTREM-1: soluble TREM-1; TNF: tumor necrosis factor; VEGF: vascular endothelial growth factor.

**Supplementary Table S8. Demographic and clinical features of HC and patients with SLE from ImmuNexUT Database in Figure 4.**

|  | **Cluster 1**  **(n = 4)** | **Cluster 3**  **(n = 3)** |
| --- | --- | --- |
| Age (years; mean [SD]) | 37.20 (14.24) | 47.33 (18.61) |
| Female (%) | 5/5 (100) | 2/3 (66.67) |
| CRP (mg/dL; median [IQR]) | 0.06 (0.02 – 0.09) | 2.89 (1.67 – 6.25) |
| Anti-dsDNA Ab  (IU/mL: mean [SD]) | 164.1 (151.8) | 2.5 (0.5) |

Anti-dsDNA Ab: anti-double stranded DNA antibody; CRP: C-reactive protein; IQR: interquartile range.
