# Supplementary figures and images for: Serum extracellular traps associate with the activation of myeloid cells in SLE patients with the low level of anti-DNA antibodies
Source: Sci Rep. 2022 Nov 1;12:18397. doi: 10.1038/s41598-022-23076-1 (PMC9626644; doi:10.1038/s41598-022-23076-1)

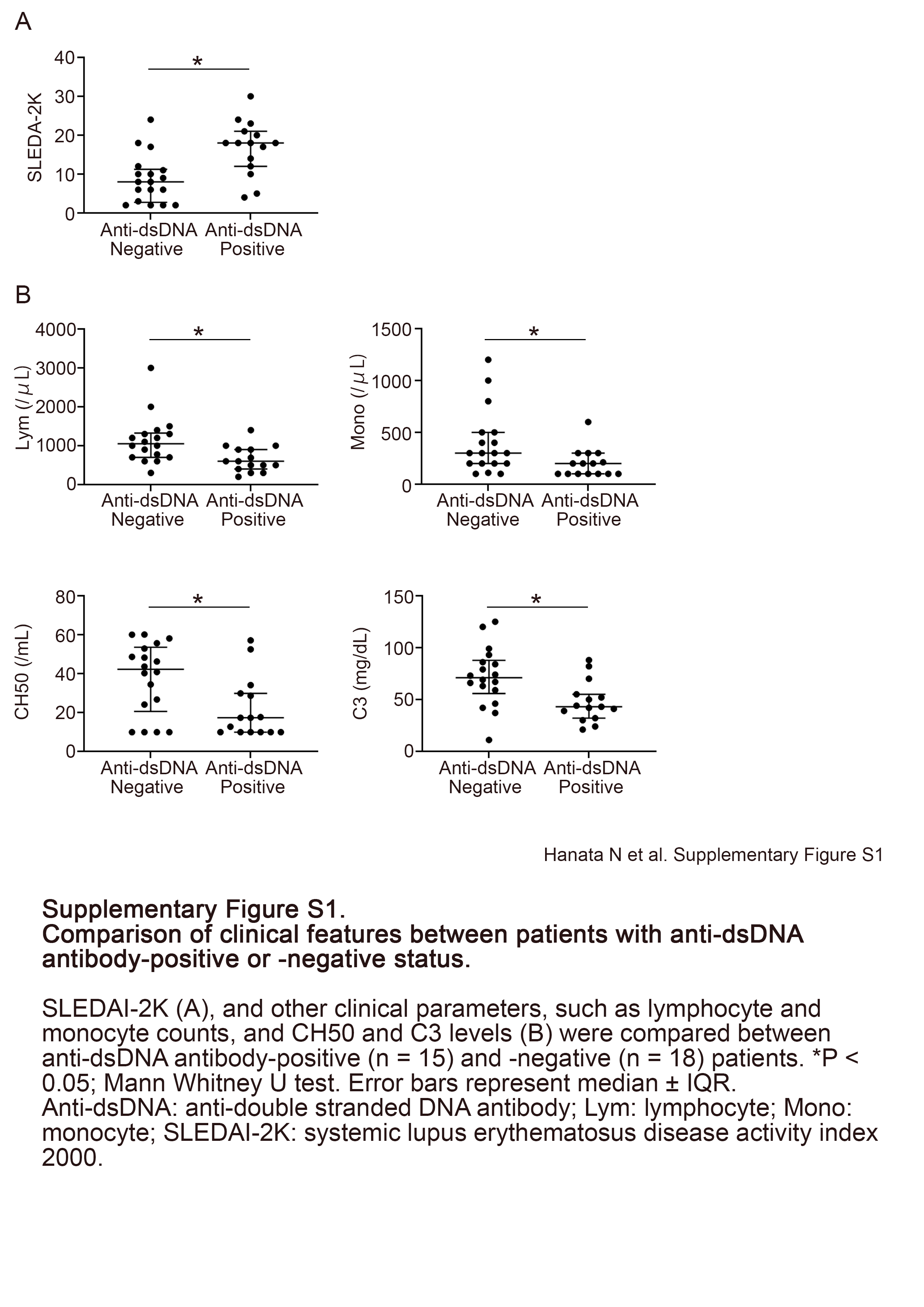

Supplement: Supplementary file 2 — Supplementary Information 2. [file 41598_2022_23076_MOESM2_ESM.tif]

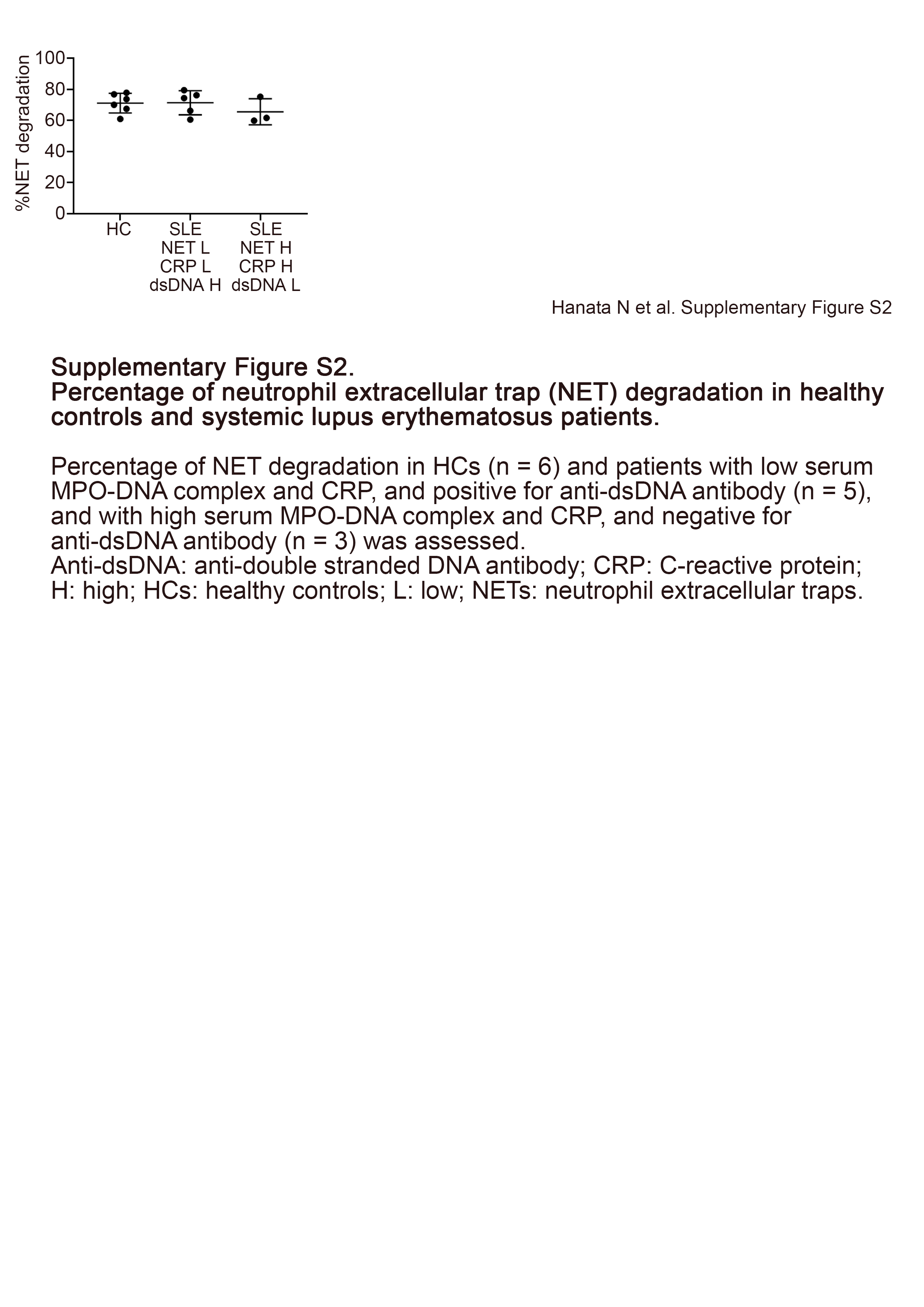

Supplement: Supplementary file 3 — Supplementary Information 3. [file 41598_2022_23076_MOESM3_ESM.tif]

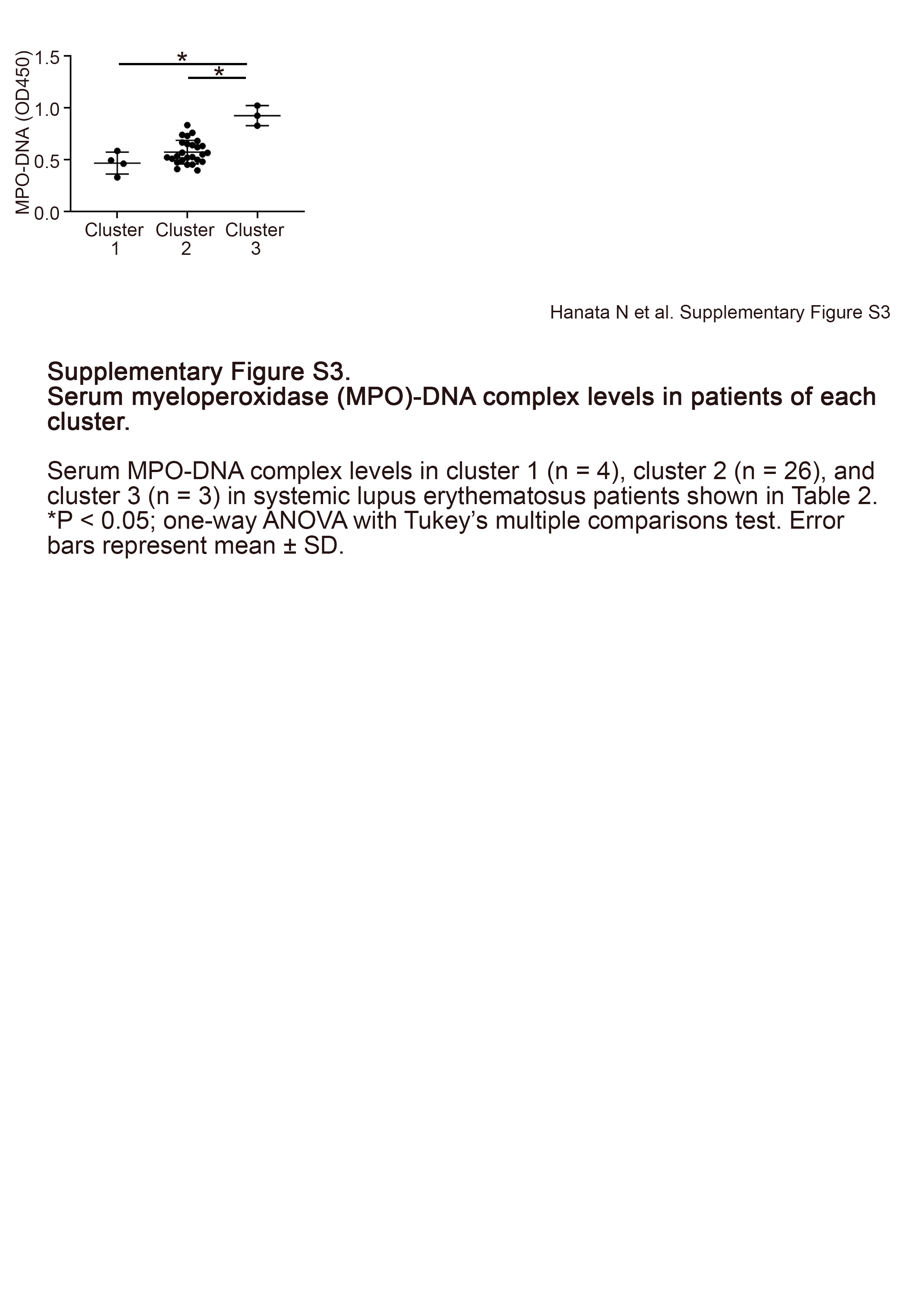

Supplement: Supplementary file 4 — Supplementary Information 4. [file 41598_2022_23076_MOESM4_ESM.tif]

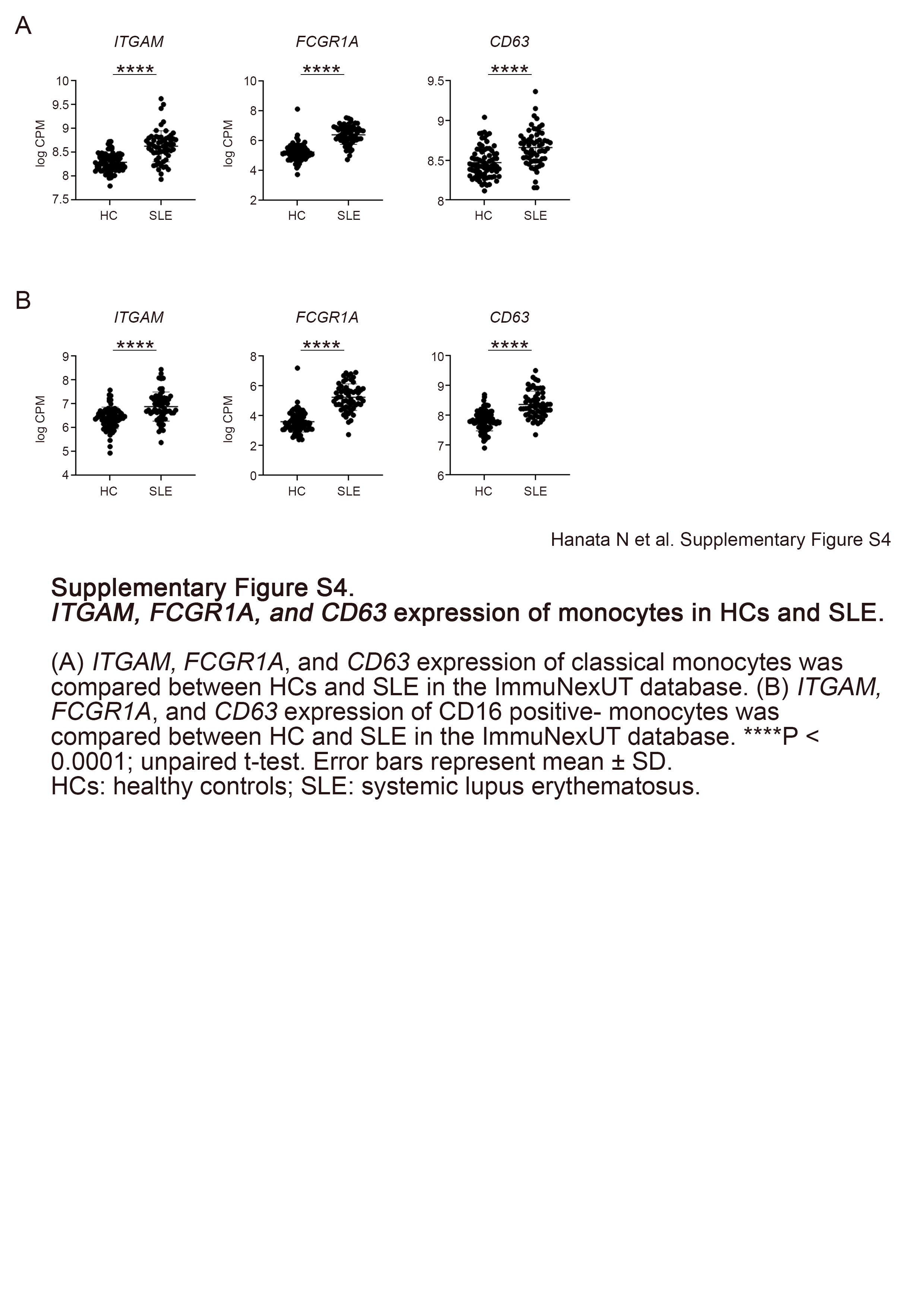

Supplement: Supplementary file 5 — Supplementary Information 5. [file 41598_2022_23076_MOESM5_ESM.tif]

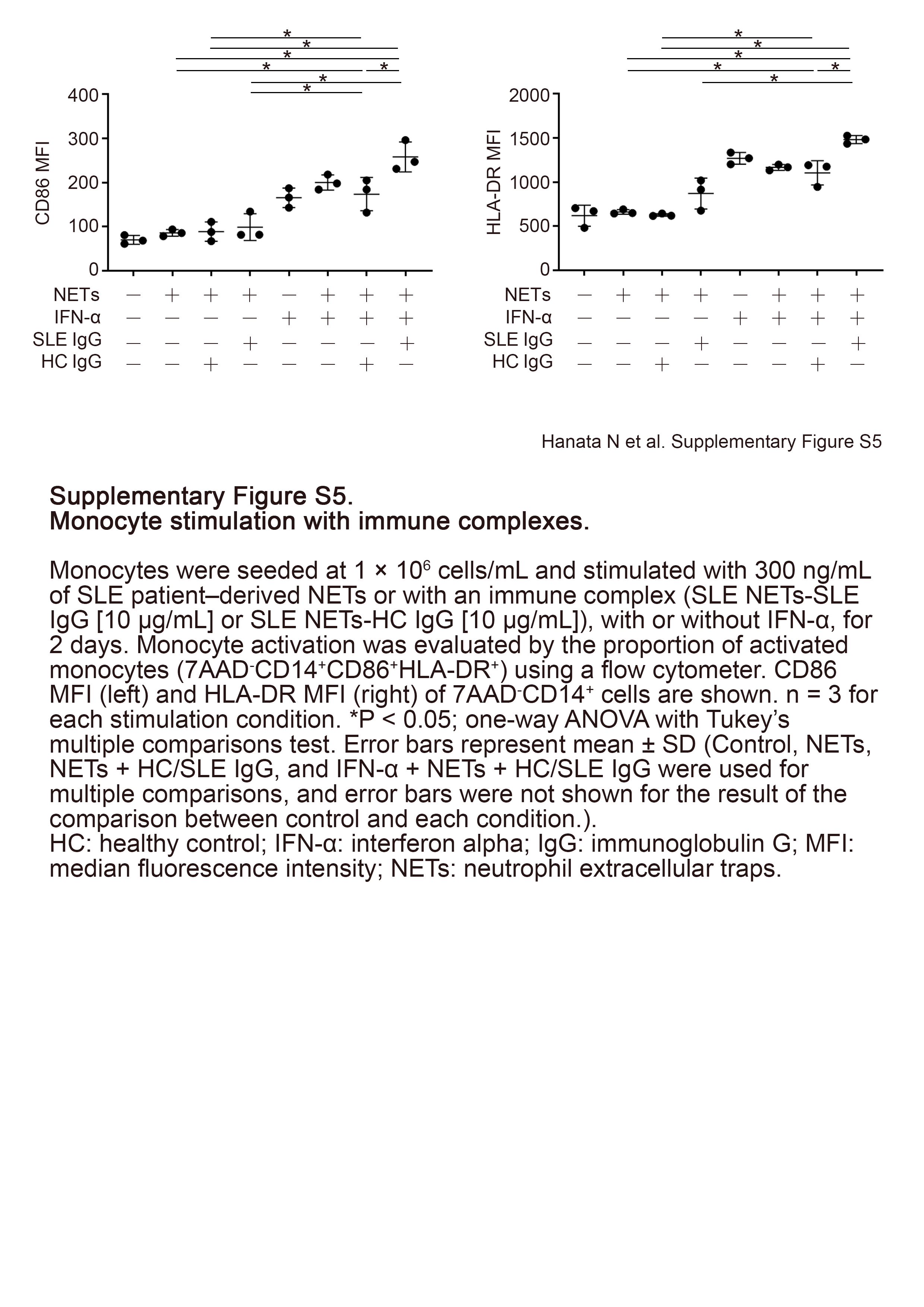

Supplement: Supplementary file 6 — Supplementary Information 6. [file 41598_2022_23076_MOESM6_ESM.tif]
